# Germs on a Plane: The Transmission and Risks of Airplane-Borne Diseases

**DOI:** 10.1177/0361198118799709

**Published:** 2018-12

**Authors:** Nereyda L. Sevilla

**Affiliations:** 1 George Mason University, Fairfax, VA

## Abstract

This research explored the role of air travel in the spread of infectious diseases, specifically severe acute respiratory syndrome (SARS), H1N1, Ebola, and pneumonic plague. Air travel provides the means for such diseases to spread internationally at extraordinary rates because infected passengers jump from coast to coast and continent to continent within hours. Outbreaks of diseases that spread from person to person test the effectiveness of current public health responses. This research used a mixed methods approach, including use of the Spatiotemporal Epidemiological Modeler, to model the spread of diseases, evaluate the impact of air travel on disease spread, and analyze the effectiveness of different public health strategies and travel policies. Modeling showed that the spread of Ebola and pneumonic plague is minimal and should not be a major air travel concern if an individual becomes infected. H1N1 and SARS have higher infection rates and air travel will facilitate the spread of disease nationally and internationally. To contain the spread of infectious diseases, aviation and public health authorities should establish tailored preventive measures at airports, capture contact information for ticketed passengers, expand the definition of “close contact,” and conduct widespread educational programs. The measures will put in place a foundation for containing the spread of infectious diseases via air travel and minimize the panic and economic consequences that may occur during an outbreak.

The risk of international spread of diseases has increased because of the advances in technology that have made global air travel a daily occurrence. More than 4.5 billion individual air journeys are made every year, increasing the risk of disease spread beyond the local area as passengers travel from coast to coast and continent to continent (Figure 1) (*1, 2*).

**Figure 1.**
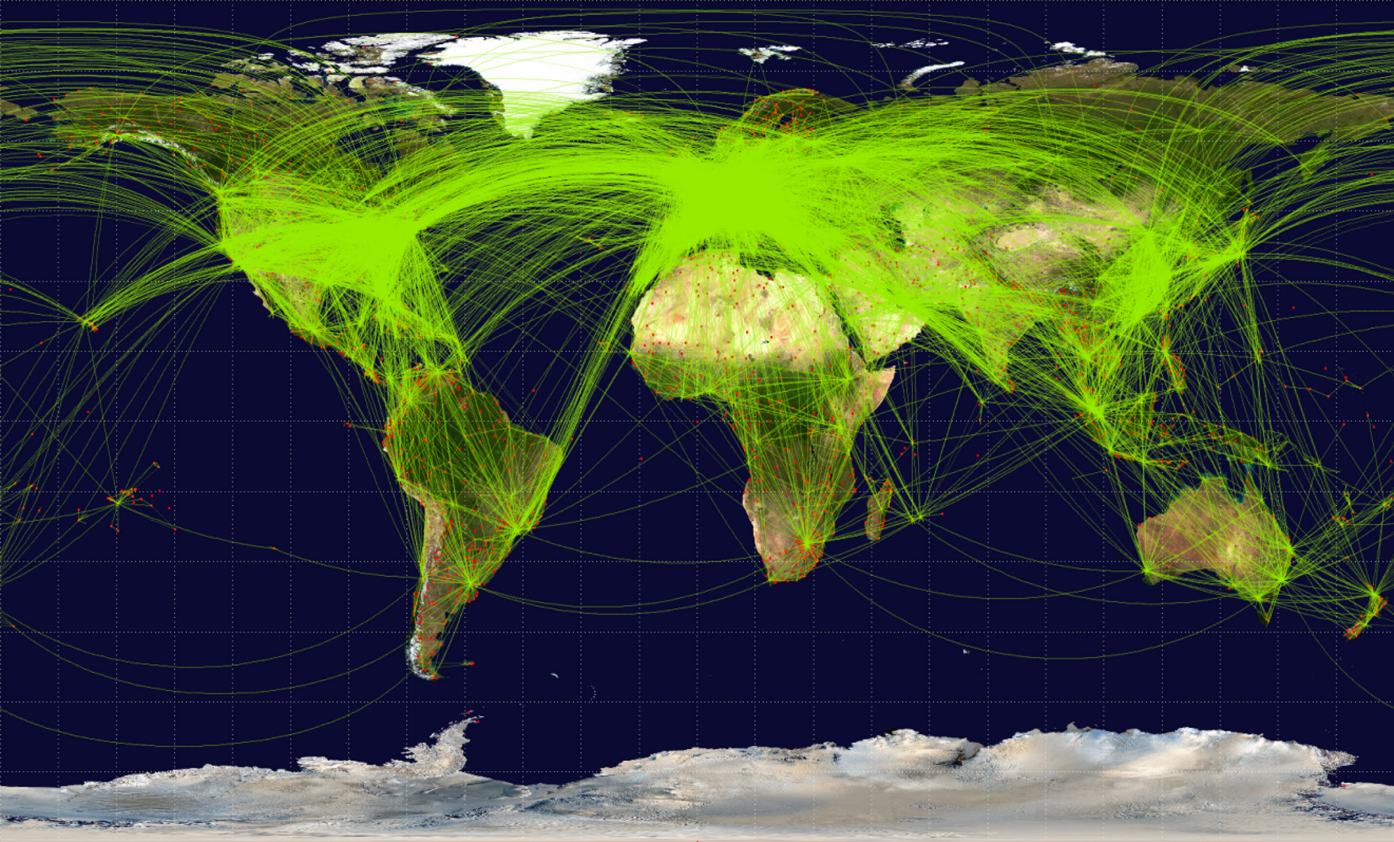
Global air travel routes.

In the twenty-first century, air travel provides new means for diseases to spread internationally at extraordinary rates because people have the ability to jump from country to country in hours. This was evident in the 2003 severe acute respiratory syndrome (SARS) pandemic that killed over 800 people across 37 countries and the 2009 influenza H1N1 epidemic that affected over 200 million individuals. By the time travel alerts and health recommendations were announced, the diseases had already reached rampant proportions, costing thousands of lives and billions of dollars. It is incumbent on scientists to develop tools that will track and predict disease spread and identify interventions that can be executed in a timely and effective manner. Plans must be based on technical and scientific knowledge of the vectors involved and the characteristics of the emerging infectious diseases.

## Methodology

This research used a mixed methods approach to evaluate the impact of air travel on the spread of infectious diseases and the effectiveness of public health strategies to mitigate disease outbreaks. A tool known as the Spatiotemporal Epidemiological Modeler (STEM), developed by IBM as an open-source program, was used to simulate four disease outbreaks in the United States, specifically SARS, H1N1, Ebola, and pneumonic plague (Table 1). Table 1 depicts the biological characteristics of the four diseases. It is important to note that SARS, H1N1, and pneumonic plague can all spread from person to person by air from coughing. Ebola is spread by the transfer of bodily fluids from person to person (e.g., sex). Another point of significance is that although pneumonic plague is extremely deadly—it kills a person in one to three days—opportunities for contagion are, therefore, limited. SARS and H1N1 have lower mortality rates, but a contagious person can spread either disease for several weeks.

**Table 1.** Biological Characteristics of Disease of Interest

	SARS	H1N1	Ebola	Pneumonic plague
Family	Coronavirus (virus)	Orthomyxoviridae (virus)	Filoviridae (virus)	Enterobacteriaceae (bacteria)
Year of outbreak	2003	2009	2014	Hypothetical
Method of transmission	Person to person by air (e.g., coughing) and fecal–oral transmission (e.g., putting dirty hands near mouth)	Person to person by air (e.g., coughing)	Direct person-to-person contact through bodily fluids (e.g., sex)	Person to person by air (e.g., coughing)
Asymptomatic transmission possible?	Low possibility	Yes	No	No
Reproductive ratio (average # of new cases generated by each case) – R_o_	2–3	1.4–3.5	1.5–2.0	1.3
Case fatality rate	13% <60 yrs 43% >60 yrs	0.01%–0.3%	50%–90%	50% (if untreated, 99%)
Symptoms	Fever, general flu-like symptoms, muscle pain	Sudden fever, body aches (joints and throat), coughing, sneezing, extreme chills, fatigue, nasal congestion	Severe frontal and temporal headache, aches and pains, fever progressing to watery diarrhea, abdominal pain, nausea, vomiting	Sudden headaches, chills, malaise, and increased respiratory and heart rates progressing to cough and fever
Treatment or vaccine	Oseltamivir, supportive care, experimental vaccine	Oseltamivir, flu shot	Supportive care, experimental vaccines	Antibiotics effective if within 24 hours of symptoms
Incubation period	1–14 days	2–6 days	2–21 days	2–4 days
Duration of illness	2–4 weeks	1–2 weeks	10–20 days	Death if untreated in 1–3 days

**Figure 2.**
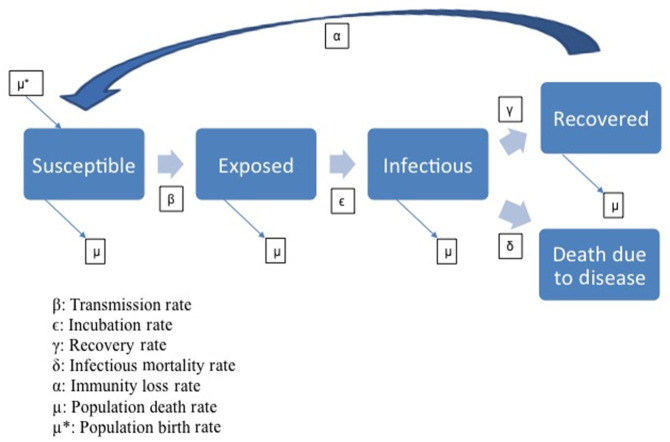
SEIR compartment model.

STEM is a multidisciplinary, collaborative modeling platform that uses compartment theory to simulate the spread of disease (*3*). The open-source characteristics of the system allow researchers to compare, refine, and validate different scenarios as well as add data such as disease characteristics. STEM provides the built-in statistics such as county and country boundaries, transportation networks, air travel information, and environmental conditions. The equations used within the compartment model provided the foundation for the model that would be used to compare the threat of disease spread while holding many characteristics constant. In compartment models, each person in a population is accounted for in a compartment, and no one person may be in more than one compartment at any given time. All the diseases of interest for this research used an “S”– “E”– “I”– “R” (SEIR) model, in which each person in a population is in one of four states: susceptible (S), exposed (E), infectious (I), or recovered (R) (Figure 2). The disease models can be configured with known disease characteristics, including infection rates, incubation period, and mortality rates. These variables can also be changed during the course of the scenario to depict possible mutations. Transitions between compartments are driven by differential equations (Figure 2). The vectors of disease that affect the transmission of an infectious disease, such as motion of individuals, ground travel, air travel, and social gatherings, can also be modeled.

The simulations in this research forecast the natural flow of disease with air travel starting with one or 10 infected individuals traveling from New York City. By applying the lessons of real-world outbreaks, public health officials can make informed decisions as to the best interventions and communication methods should a disease outbreak occur.

## Results

The results illustrate the impacts of hypothetical outbreaks in the United States, starting in New York, beginning in September and lasting for 6 months. These simulated cases of SARS, H1N1, Ebola, and pneumonic plague are all based on known infection parameters, such as basic reproductive ratio (R_o_), transmission rate (β), incubation rate (ϵ), recovery rate (γ), and infectious mortality rate (δ), and are based, in turn, on data from previous outbreaks, historical epidemiological numbers, scientific evidence, and biological characteristics as outlined in Table 1.

The models of SARS, H1N1, Ebola, and pneumonic plague mirror the actual historical outbreaks in the United States and are assumed to follow similar patterns if another outbreak were to occur (Table 2). The results indicate that SARS and H1N1 have a much greater impact in relation to infections and deaths than Ebola or pneumonic plague. All of these results assume a natural flow of disease based on biological characteristics and population movement and do not include or assume any public health interventions.

**Table 2.** Simulated and Actual Disease Cases

Simulated and actual cases in the United States		
Simulated diseases (1 or 10 initial cases)	Total infected (% of the population)	Total deaths
SARS – 1	4,042 0.0014%	572 0.0002%
SARS – 10	7,279 0.0026%	1,000 0.0004%
H1N1 – 1	5,617,702 1.9962%	5,374 0.0019%
H1N1 – 10	18,363,550 6.5253%	18,856 0.0067%
Ebola – 1	5 <0.0001%	1 <0.0001%
Ebola – 10	43 <0.0001%	19 <0.0001%
pneumonic plague – 1	1 <0.0001%	1 <0.0001%
pneumonic plague – 10	25 <0.0001%	13 <0.0001%
Historical diseases	Total infected	Total deaths
2003 SARS United States	27 <0.0001%	0 0%
2003 SARS Canada	438 0.0015%	44 0.0001%
2003 SARS China	5,327 0.0004%	349 <0.0001%
2009 H1N1 United States (Apr–Oct 2009)	~22 million 7.8174%	~3,900 0.0014%
2014 Ebola United States	4 <0.0001%	1 <0.0001%
2015 pneumonic plague United States	11 <0.0001%	3 <0.0001%

In comparison with the historical accounts of SARS, H1N1, Ebola, and pneumonic plague, the hypothetical scenarios of each of the disease spreads follow similar patterns and results. The model shows that an outbreak of SARS in the United States would cause approximately four to seven thousand cases and 500 to 1,000 deaths. Although the United States experienced a small number of SARS cases but no SARS deaths, its neighboring country of Canada did confront 438 cases and 44 deaths (*4*). Of the four diseases, H1N1 has the most impact on the population according to the model, with 5 to 18 million people affected and five to 18 thousand deaths. During the 2009 H1N1 outbreak, the United States experienced approximately 22 million disease cases and approximately 4,000 deaths during a 6-month period (*5*). During the Ebola crisis in 2014, the United States had two imported cases of Ebola, and two locally acquired cases by medical professionals. Specifically two nurses that directly treated the first case of imported Ebola were the only locally transmitted cases of Ebola in the US. No cases of Ebola were acquired through air travel. Finally, the United States experiences approximately 10 to 15 cases of pneumonic plague per year, but has not had a person-to-person spread of pneumonic plague since 1925 (*6*). There has never been a case of pneumonic plague associated with air travel. The model shows very small numbers of individuals infected by and dying because of pneumonic plague and Ebola as compared to SARS or H1N1 should outbreaks occur.

The comparative results of each of the four modeled diseases along with the historical accounts show the importance of the disease characteristics and the impact of the infection rate. A disease that has a long period of illness, such as SARS or H1N1, is expected to cause a higher natural spread than diseases in which the period of illness is brief. In cases in which the period of illness is short, such as pneumonic plague, those individuals affected do not have the same opportunities to infect others as do those people harboring diseases with a longer period of illness.

### Limitations

The technology exists to rapidly build many new models of infectious diseases. A “good approximation” requires not only knowledge of the disease parameters themselves, but also an understanding of the most important disease vectors. The SEIR compartment model used in this research compared one disease to another while controlling for similar variables such as environmental and population data. However, in actuality, the reproductive rate of a disease, the incubation rate, recovery rate, and mortality rate can all vary based on socioeconomic factors, gender, and age.

This research focused on an average population in the United States, on the impact of air travel, and on the policy for controlling the spread of disease, based on typical disease characteristics. For air travelers, exposure is often related to their purpose for travel, that is, visiting friends and interacting with the population, or pure tourist or business travel, in which exposure may be less. Infectious individuals in the model travel at the same rate as non-infectious individuals, which may not be true under real travel conditions (*7*). Sick individuals may elect to stay home and this would reduce the rate of infection and help minimize the disease outbreak. However, the model does normalize air travel for individual diseases and allows for the long-distance jump from one coast to another (*8*). These limitations in the overall computer modeling are minor because the model provided for control of travel among the different diseases depicted. The outcomes are seen in the trends as one disease is compared to another, but the numbers of infectious or deceased individuals should not be used as absolutes.

Furthermore, the models assume the pathogens of interest mirror previous outbreaks with known disease features. If the genetics of any of the diseases are changed by natural mutation or by intentional genetic manipulation, the outcomes of disease spread may change dramatically. The manipulation of the disease characteristics may change the recommendations if the disease spread or mortality rate is greater than previously observed. This research assumes no manipulation or genetic mutation has occurred.

## Recommendations

In the event of an infectious disease epidemic, the world must act swiftly and decisively, and unite to form a cohesive plan based on science to limit the spread of disease, thus reducing economic costs and psychological impacts. Officials must implement measures to track the pattern of disease spread as soon as the threat is identified, especially if the disease of concern, for example SARS or H1N1, has a high transmission rate. Therefore, when an infectious disease outbreak occurs, it is imperative to have appropriate travel and communication policies in place. In cases involving Ebola and pneumonic plague, efficient and cost-effective measures will limit apprehension and minimize the economic consequences.

This research outlined physical and policy recommendations for implementing effective measures in response to a disease outbreak; all are rooted in the education of the public. Mitigation measures are also outlined in the *Airport Cooperative Research Program Report 91: Infectious Disease Mitigation in Airports and on Aircraft*, which includes 24 recommended actions for buildings, airplanes, and people (*9*). Many of the recommendations in this report are reiterated in this research; six specific recommendations are expanded upon below. Research showed that simple measures may improve global travel health. Their implementation has the potential to limit death, minimize infections, decrease economic impacts, and curtail fear.

### Recommendation #1: Expand the Definition of “Close Contact”

The World Health Organization (WHO) defines close contact as the same row plus two rows ahead and two rows behind the identified ill individual (*10*). Previous research indicated that the risk of transmission while traveling in an airplane is very low. (*10*[Bibr bibr11-0361198118799709][Bibr bibr12-0361198118799709]–*13*) An airplane has a protective mechanism during flight that includes air being constantly circulated through high-efficiency particulate air filters and mixed with outside air. However, close contact with ill individuals in airports may prove to be a more likely route of transmission than contact on the airplane. Airport scenarios include and are not limited to: extended close contact while seated prior to boarding; close contact with individuals at neighboring gates traveling to other destinations; delays that necessitate being near to other travelers in lines at check-in, security screening, restrooms, and concessions; and close contact in a confined jetway space while waiting to board. Because individuals are encouraged to arrive at the airport hours before a flight, they may spend more time at the airport than on the airplane itself. During this time, an individual may be in close contact with many more individuals outside of an airplane than while flying. In such cases, non-infected individuals may be exposed to infectious individuals. Policies must take into account all the individuals on an airplane and within the timeframe of the exposure window at the airport. It will take the cooperation of public health officials and government agencies to recognize the science of disease spread and respect the privacy of individuals and, therefore, communicate to the traveling population in the most appropriate way the possible risk of disease infection, thus minimizing its spread.

### Recommendation #2: Health Contact Information Requirement on all Air Travel Ticket Purchases

The use of a passenger manifest to track down and trace passengers who may have been affected by a diseased individual is not only very resource intensive but it may also miss a large population of other close contacts outside of the airplane. To improve contact information across airport populations, additional contact requests should be required on all tickets (Table 3).

**Table 3.** Example of Disease Notice on Air Travel Ticket Purchases

Notification of disease in travel areas, airports, or on airplanes
Do you wish to be notified of an outbreak of any infectious disease of concern* reported in the travel area, airport, or on board the airplane? □ Yes □ No
If yes, contact information must be provided:
□ Text:
□ E-mail:
□ Voicemail:
□ Other, please provide complete contact information:
All current travel alerts may be found at: http://wwwnc.cdc.gov/travel/notices/.
*Infectious diseases of concern include: (1) cholera, (2) diphtheria, (3) infectious tuberculosis, (4) plague, (5) smallpox, (6) yellow fever, (7), viral hemorrhagic fevers, (8) SARS, and (9) flu that can cause a pandemic.
Please consult the CDC website to determine if an outbreak is relevant to your travels.

Efficient capture of passenger contact information is a necessary response to a disease outbreak. If the ticketed passengers at the airport have already given their contact information, a widespread communication effort can be made to reach all those with tickets at the airport during the time of the infectious exposure.

### Recommendation #3: Expand Passenger Airport and Pre-Boarding Self-Sanitizing Measures

Filters on airplanes serve as a baseline preventive measure against in-flight disease transmission. However, their effectiveness is greatly increased when passengers themselves practice infection control techniques. Airport announcements and visual aids about disease preventive measures displayed around the terminal provide instant reminders and education. This just-in-time education would be fresh in passengers’ minds and emphasize to them that they will be at close quarters with other travelers at the gate, on the jetway, during the boarding process, and during flight. Airlines and airports should make hand sanitizers readily available, especially at restroom entrances, at the gate area before boarding an airplane, at all food court locations, at the entrances of all the airport stores, in all the lounge areas, and randomly around all the corridors. During embarkation, the gate officials should be encouraging everybody to use hand sanitizers and expand personal space on the jetway. These immediate instructions would increase compliance. At the same time, airport announcements should remind passengers to cover their coughs with hands or elbows. Additionally, airlines could provide personal handwipes, allowing passengers to clean their hands and other areas that harbor germs, such as fold-down trays. Everybody should carry out the preventive measures. This continuous emphasis on good habits may start a culture trend and natural habit formation, not just during an outbreak, but at all times, and such low-cost preventive measures may minimize pathogen transmission.

### Recommendation #4: Enhanced Travel Alerts and Advisory Notices during Ticket Sales

The use of health statements on air travel ticket purchases is also a way of encouraging every passenger to curtail personal travel to world areas of concern as well as limit personal travel during an illness. Travel restrictions have been found to be minimally effective and must be strictly enforced to be successful. Historical data have indicated that travel restrictions may only cause a slight delay in infectious disease introduction to the United States (*10*, *14*, *15*). It is more cost effective to inform the public about the various appropriate resources available in order for them to make a more informed choice. Notices of health concerns should be displayed during online ticket purchase or at a ticket counter and relayed over the phone (Table 4).

**Table 4.** Example of a Health Notice Given during Air Travel Ticket Purchase

WARNING: TRAVEL AREA HAS HEALTH ALERT FOR PNEUMONIC PLAGUE!
All current disease travel alerts and advisory notices may be found at: http://wwwnc.cdc.gov/travel/notices/.
Do not travel if suspected of carrying a disease of concern.*
*Infectious diseases of concern include: (1) cholera, (2) diphtheria, (3) infectious tuberculosis, (4) plague, (5) smallpox, 6) yellow fever, (7), viral hemorrhagic fevers, (8) SARS, and 9) flu that can cause a pandemic.
Please consult the CDC website to determine if an outbreak is relevant to your travels.

Travel alerts and advisory notices in combination with educating travelers about proper preventive measures would reduce the risk of infection. The advisory notices should come from major health agencies such as the WHO and the Center for Disease Control and Prevention (CDC). A strong message may encourage the public to further find information about a disease and their risk of infection. In the event of an infectious disease outbreak, clear and concise travel alerts will help in reducing the spread of infection through air travel. Each of the agencies should declare a notice when scientifically applicable and disseminate that information through various social media outlets, thereby enhancing those messages given at the airport and accompanying each ticket sale.

### Recommendation #5: Limited, Announced, Random Temperature Checks

Targeted entry and exit procedures have been shown to have a limited effect on the spread of disease (*16*, *17*). However, in a dire situation, these very costly procedures may be a measure of last resort to prevent a deadly disease from spreading. It must be noted, however, that the deadliness of the disease must outweigh the economic and political impact of such a decision. It would also only be effective with the complete cooperation of all international airlines and country public health officials.

Limited and announced, yet random temperature checks during an outbreak may deter ill individuals from traveling. Thermal screenings themselves may not be cost effective in halting the spread of disease. However, the threat of entry and exit procedures may be sufficient to deter as many individuals from traveling as would a large-scale thermal screening effort. If an infectious disease such as SARS is detected in New York, then any airplane from New York airports may be subjected to random screening tests. If, during the ticketing process, a passenger is alerted to the random check, that person may decide not to purchase the ticket or change their travel plans (Table 5).

**Table 5.** Example of a Thermal Screening Alert on an Air Travel Ticket

**ALERT** **SARS Outbreak Concern in New York City** **Random Thermal Screening Instituted**
Your travel plans involve an airplane flying from New York City.
You may be subjected to random thermal screening due to a suspected outbreak of SARS. If you suspect you are ill, do not travel and delay your travel until fever symptoms have subsided. Please consult a doctor if you have concerns about disease exposure. Do you understand that you may undergo thermal testing during your travels? □ Yes □ No
Please consult the CDC website for more travel information: http://wwwnc.cdc.gov/travel/notices/.

Similarly, during check-in, if an outbreak is confirmed in a travel area, the government and airlines should work together to ensure passengers are incentivized to rethink their travel plans. These measures include a full refund or changes in dates without penalty. The same should be true if passengers find themselves ill. To prevent the spread of disease, passengers should be allowed to change their plans without penalty after consultation with and providing documentation from authorized physicians. Working with public health officials, members of the public may take personal responsibility and postpone travel until the threat of contagion has passed.

### Recommendation #6: Specific Crisis Communication

The basis for early containment procedures is constant and relevant communication. Early messages during a disease crisis must educate the public on the medical threats during travel. Education of the public is the crucial foundation for and probably the most cost-efficient and effective way of preventing the spread of disease in the first place and slowing the spread if it should start.

In the event of an outbreak, communication barriers arise when individuals take advice from a source of their choosing, especially if that source is not an expert. Emergency response officials must provide consistent, accurate, and simple information that can help the public prevent transmission of infectious disease, detect symptoms, and seek treatment so that uncertain individuals do not look elsewhere for information. Messages should provide pertinent and applicable information such as general prevention methods, the differences in the presentations of various illness, and treatment methods.

To guide public health officials, the WHO produced a handbook entitled *Effective Media Communications during Public Health Emergencies* (*18*). This has been the guiding foundation of many instructions issued by countries and local organizations in the midst of a crisis. Critical tips for effective communication include:

#### Provide Early and Constant Communication and Avoid Rumors from Non-Credible Sources

Individuals will start doing their own research when the rumor of a disease outbreak begins to circulate. Efforts should be made to ensure credible websites and information sources are prominently advertised. Individuals should be steered toward websites such as CDC Travelers’ Health (https://wwwnc.cdc.gov/travel), U.S. Department of State (sections Passports, and International Travel) (https://travel.state.gov/content/travel/en.html), and WHO International Travel and Health (http://www.who.int/ith/diseases/en/). Potential passengers may also start looking at travel websites, airline home pages, or other sources of information, official or unofficial. The timeliness with regard to how and where messages are conveyed may help lessen panic. However, studies have shown that under stress, individuals usually compare current messages to the first pieces of information processed, even if later messages are more accurate (*19*). It is imperative that scientifically validated messages are posted in multiple locations, especially on common travel websites and in news outlets.

#### Simple, Honest Communication Statements Created from Message Maps

The CDC recommends constructing message maps to convey standardized messages that target specific audiences. A message map packages important facts about the disease and the risks of disease spread in simple sentences. Public relations offices could use message maps to guide their announcements so that honest, trustworthy, and relevant information can be conveyed to potential travelers. As an example, because pneumonic plague already has a fearsome reputation as the “Black Death,” it is essential to provide accurate and easily accessible information in a message map (Table 6).

**Table 6.** Example of a Pneumonic Plague Message Map

Stakeholder: Public Question: Will I get pneumonic plague on an airplane when flying to or from an outbreak area?		
Key message 1	Key message 2	Key message 3
Pneumonic plague is extremely difficult to contract person to person	Infection rate is extremely low	It is very difficult to spread pneumonic plague coast to coast
Supporting information 1–1	Supporting information 2–1	Supporting information 3–1
85% of pneumonic plague (also known as bubonic plague) cases are caused by transmission of the disease by rodents, and the disease does not spread easily from human to human	A person is only infectious for 1–3 days	The United States reports approximately 25 cases of pneumonic plague a year. There has not been a sustained outbreak for nearly a century.
Supporting information 1–2	Supporting information 2–2	Supporting information 3–2
Pneumonic plague spread from person to person usually occurs after long periods of close contact, for example, in relation to caregivers or medical professionals	A person becomes ill very rapidly and it would be difficult to travel while experiencing symptoms	A disease with such a low infection rate cannot sustain a US-wide outbreak
Supporting information 1–3	Supporting information 2–3	Supporting information 3–3
A person is not infectious without symptoms	A person suffering from pneumonic plague would be easier to spot than someone suffering from another disease due to the plague’s rapid progression and severity	Previous large numbers of pneumonic plague deaths were due to unsanitary conditions, lack of modern medicine and antibiotics, and large numbers of rodents

In the event of a disease outbreak, it is as important to state what is unknown as it is to state what is actually known. Public health and government officials maintain credibility when they explain what factors are still unknown instead of stating what the public may want to hear. More panic may be created if a wrong message is conveyed. Even if officials present new and accurate information later, their credibility could have been destroyed by not being honest earlier about the unknowns. Therefore, it is best that public officials state “We don’t know,” because an educated guess may backfire in the long run. An untrue statement will become a roadblock to any future truthful or useful statements. All statements need to be verified by scientific evidence from validated sources.

Furthermore, it is also important that officials advise the public when further information will be available, how those messages will be presented, and when official social media and websites will be updated. The public will look for new information if told when and where to find it in its most up-to-date form. However, it is key not to give false hope or guarantee victory unless a victory is 100% guaranteed. If the source of the disease has been contained and secondary infections isolated, then the public can be reassured.

Inaccurate information, and promises broken early on may worsen a crisis. The public will lose all trust if early in a crisis the officials prove to be wrong and may turn to anyone who appears to be right even though those individuals are unqualified. Public officials need to establish their expertise and credibility as soon as possible.

#### Empower the Public

The public must be shown how their actions will help prevent the spread of disease. Educational programs should include: the necessity of coughing into a hand/elbow during a flight and the use of hand sanitizer. If officials lead by example, it may create a natural habit pattern. When actions of trusted individuals mirror the health guidelines, the public will be more accepting of the underlining message. Studies have shown that if the public can take actions during a crisis it can help restore a sense of control and overcome feelings of hopelessness and helplessness (*19*).

Different media outlets should disseminate the same messages to accommodate the various generational, political, and social differences and, particularly, these should be tailored to their local population. Despite the differences in media markets, all should broadcast the same consistent message. National and local leaders, including educators and religious officials, can also disseminate simple preventive and basic infection control measures. For example, teachers can remind students to wash their hands before lunch periods. These simple messages will have a lasting impact, reduce stress and, ultimately, lessen the spread of disease.

### Discussion of Results

The spread of disease during travel is a concern; however, diseases such as SARS and H1N1, which have a high transmission rate, will have a greater impact on the population more in relation to infections and deaths than Ebola or pneumonic plague, even though the latter two may present more of a psychological panic. Efforts should be made to contain the disease and communicate the threat based on scientific evidence. The airport itself may be more of a location for transmission of disease because individuals wait in the same ticketing lines, gates, and jetways as infected passengers. The recommendations presented are relevant to passengers who are in the vicinity of infected individuals at the airport and not just traveling on an airplane.

Historical examples and models show that the spread of SARS and H1N1 is significantly affected by air travel. Because of the advancements in travel technology and engineering, humans can reach any part of the globe within the incubation time of most diseases, allowing travelers to unintentionally carry a disease. Ebola and pneumonic plague have short illness durations with a high probability of death, so the risk of transmission is low and the outbreak is not sustainable without further incubators. However, all public health professionals should be prepared to deal with a disease that may not be endemic to an area. Because of air travel, all areas of the Earth are susceptible to all types of diseases.

In each of the recommendations, the component of public education is of the utmost importance. The scientific and medical advancements in disease prevention and treatments mean that many infectious diseases are no longer seen as serious biological threats. However, many diseases, such as pneumonic plague, remain as serious psychological threats. Public health officials need dedicated resources with which to create communication strategies to reduce the psychological fallout from a disease outbreak. This, in turn, minimizes the economic consequences from such an outbreak.

To contain the outbreak of diseases, aviation and public health authorities should establish preventive infectious disease measures at airports, capture contact information for ticketed passengers, expand the definition of “close contact,” and conduct widespread educational programs. These measures will put in place a foundation for containing the spread of infectious diseases, minimize the panic, and reduce costs that may occur during an outbreak.

## References

[1] Kirwan D. Global Health: Current Issues, Future Trends and Foreign Policy. Clinical Medicine, Vol. 9, No. 3, 2009, pp. 247–253. 10.7861/clinmedicine.9-3-247 PMC4953612 19634388

[2] OpenFlights.org. Airport, Airline and Route Data. http://openflights.org/data.html. Accessed June 2017.

[3] Sevilla N. Open Source Disease Modeling: A Tool to Combat the Next Pandemic. Global Biodefense, 2016. https://globalbiodefense.com/2016/01/28/open-source-disease-modeling-a-tool-to-combat-the-next-pandemic/. Accessed June 2017.

[4] National Advisory Committee. Renewal of Public Health in Canada: Learning from SARS. National Advisory Committee on SARS and Public Health, Ottawa, Ontario, Canada, 2003.

[5] Centers for Disease Control and Prevention. H1N1 Flu. CDC Estimates of 2009 H1N1 Influenza Cases, Hospitalizations and Deaths in the United States, April–October 17, 2009. http://www.cdc.gov/h1n1flu/estimates/April_October_17.htm. Accessed June 2017.

[6] U.S. Army Medical Research Institute of Infectious Diseases. Medical Management of Biological Casualties Handbook, 7th ed. U.S. Army Medical Research Institute of Infectious Diseases, Fort Detrick, MD, 2011.

[7] Johansson M. On the Treatment of Airline Travelers in Mathematical Models. PLoS One, Vol. 6, No. 7, 2011, p. e22151. 10.1371/journal.pone.0022151 PMC3143116 21799782

[8] Lessler J. The Cost of Simplifying Air Travel When Modeling Disease Spread. PLoS One, Vol. 4, No. 2, 2009, p. e4403. 10.1371/journal.pone.0004403 PMC2633616 19197382

[9] Environmental Health & Engineering, Inc. ACRP Report 91: Infectious Disease Mitigation in Airports and on Aircraft. Transportation Research Board of the National Academies, Washington D.C., 2013.

[10] Gaber W. Screening for Infectious Diseases at International Airports: The Frankfurt Model. Aviation, Space, and Environmental Medicine, Vol. 80, No. 7, 2009, pp. 595–600. 10.3357/asem.2360.2009 19601499

[bibr11-0361198118799709] Ong R. Airline Policies and Procedures to Minimize the Spread of Disease. Presented at 89th Annual Meeting of the Transportation Research Board, Washington D.C., 2010.

[bibr12-0361198118799709] Rydock J. Tracer Study of Proximity and Recirculation Effects on Exposure Risk in an Airliner Cabin. Aviation, Space, and Environmental Medicine, Vol. 75, No. 2, 2004, pp. 168–171. 14960054

[13] Mangili A. Transmission of Infectious Diseases during Commercial Air Travel. The Lancet, Vol. 365, No. 9463, 2005, pp. 989–996. 10.1016/S0140-6736(05)71089-8 PMC7134995 15767002

[14] Cooper B. Delaying the International Spread of Pandemic Influenza. PLoS Med, Vol. 3, No. 6, 2006, p. e212. 10.1371/journal.pmed.0030212 PMC1450020 16640458

[15] Poletto M. Assessing the Impact of Travel Restrictions on International Spread of the 2014 West African Ebola Epidemic. Euro Surveill, Vol. 19, No. 42, 2014, pp. 1–6. 10.2807/1560-7917.es2014.19.42.20936 PMC4415609 25358040

[16] European Centre for Disease Prevention and Control. Infection Prevention and Control Measures for Ebola Virus Disease: Entry and Exit Body Temperature Screening Measures. European Centre for Disease Prevention and Control, Stockholm, Sweden, 2014.

[17] Wilder-Smith A. Experience of Severe Acute Respiratory Syndrome in Singapore: Importation of Cases, and Defense Strategies at the Airport. Journal of Travel Medicine, Vol. 10, No. 5, 2003, pp. 259–262. 10.2310/7060.2003.2676 PMC7107530 14531977

[18] World Health Organization. Effective Media Communications during Public Health Emergencies. World Health Organization, Geneva, Switzerland, 2005.

[19] Center for Disease Control and Prevention. Crisis and Emergency Risk Communication. U.S. Department of Health and Human Services, Washington, D.C., 2014.

